# Item

**Published:** 1899-03

**Authors:** 


### ITEM.

MESSRS. WILLIAM R. WARNER, manufacturing chemists, of Philadelphia, sustained a heavy loss by fire late in February, which destroyed their office building and retail department, at 1228 Market street. Their laboratory, however, was located in another building, which was unharmed, hence their extensive business will go on as usual.

